# Using C-Reactive Protein Measurements to Predict Treatment Failure in Native Vertebral Osteomyelitis: A Multisite Retrospective Cohort Study

**DOI:** 10.1093/ofid/ofag239

**Published:** 2026-04-22

**Authors:** Brendan Campbell, Tim Lahey, Andrew J Hale

**Affiliations:** Division of Infectious Diseases, Department of Medicine, University of Vermont Medical Center, Burlington, Vermont, USA; Division of Infectious Diseases, Department of Medicine, University of Vermont Medical Center, Burlington, Vermont, USA; Division of Infectious Diseases, Department of Medicine, University of Vermont Medical Center, Burlington, Vermont, USA

**Keywords:** antimicrobial management, C-reactive protein, epidural abscess, outpatient parenteral antibiotic therapy, vertebral osteomyelitis

## Abstract

**Background:**

The management of native vertebral osteomyelitis (NVO) can be challenging due to imperfect prognostic tools to predict treatment outcome. It is common practice to use serial serum C-reactive protein (CRP) to help decide treatment response, although limited evidence supports this practice. We evaluated whether CRP assessment predicts treatment failure in NVO.

**Methods:**

We conducted a multisite retrospective cohort study at the University of Vermont Health Network among adults with NVO managed with ≥6 weeks of antibiotics and in whom there were ≥2 documented CRP values at least a week apart. The primary outcome was microbiologic or clinical failure of antibiotic therapy within 6 months of the initial diagnosis.

**Results:**

Among 628 patients identified by the initial screening criteria, 143 met inclusion criteria. In univariate analyses, the median last CRP value differed between patients with treatment success and those with treatment failure (8.9 vs 21.0 mg/dL; *P* < .01), as did the median percent decrease from first to last CRP measurement (88.9% vs 76.9% reduction; *P* = .03). However, in multivariate analyses adjusting for *Staphylococcus aureus* infection and epidural abscess we observed only weak and nonsignificant associations between CRP values and treatment failure (odds ratios, 1.02 for median last CRP value [*P* = .06] and 0.99 for median percent difference (*P* = .11).

**Conclusions:**

Serial CRP measurements were only weakly associated with treatment failure in NVO. This common clinical practice is likely of little value.

## Teaser

Serial C-reactive protein (CRP) measurement is a common management practice in patients with native vertebral osteomyelitis. In a retrospective multisite cohort study, however, CRP values were only weakly associated with treatment failure in these patients, calling this practice into question.

The incidence of native vertebral osteomyelitis (NVO) is rising in the United States and associated with longer average hospital stays and resulting increases in healthcare costs. In addition, the substantial patient morbidity from treatment failure can result in significant complications, including neurologic dysfunction, inability to walk without assistance, bladder or bowel incontinence, and chronic pain [1–3].

The diagnosis of NVO can be challenging, given the low sensitivity of biopsy (30%–74%) [4]. Because there are no definitive methods to characterize the adequacy of patient treatment response, checking serial C-reactive protein (CRP) values is common in NVO since other modalities, such as spinal imaging, are poorly correlate with clinical status. Thus, CRP measurements are often used to gauge the likelihood of treatment failure before finalizing the treatment duration [4–6].

However, the true utility of CRP in the management of NVO is not well understood. One study suggested that CRP can help predict treatment outcomes for spinal infections, including postoperative spinal infections [7]. High CRP values at diagnosis and persistent CRP elevation after treatment have been associated with an increased risk of treatment failure, neurologic sequelae, and death [2, 3, 8, 9]. These data have been cited to support serial CRP monitoring during the treatment of NVO [10], though further assessment is needed. To guide clinical practice, we investigated the association between NVO treatment failure and both the last CRP value and the magnitude of CRP decrease from the start of treatment.

## METHODS

### Study Design

We performed a retrospective cohort study at the University of Vermont Health Network (UVMHN) hospital system, with institutional review board approval and waiver of informed consent. Our study included patients from 3 UVMHN hospitals serving >1 million patients throughout Vermont and northern New York.

### Patients

Between 1 January 2011 and 1 June 2024 we enrolled adult patients (aged >18 years) with the diagnosis of NVO (based on codes M46.20–M46.28, M46.30–M46.39, and M46.40–M46.49 from the *International Classification of Diseases, Tenth Revision*). Given the overlap in these codes between NVO and sacroiliitis, we reviewed patient records to identify and exclude patients with only sacroiliitis. The diagnosis of NVO was determined by positive blood or vertebral disk culture that was correlated with magnetic resonance imaging or computed tomography (CT) imaging findings of vertebral osteomyelitis or diskitis. The diagnosis of culture-negative NVO was based on the clinical judgment of an infectious diseases physician. The specific antibiotic choice was determined by the treating infectious diseases physician and was not standardized. Only the first episode of NVO for a given patient was included. We collected data on patient age, sex, race, medical comorbid conditions, the spinal segment involved, complications of NVO including epidural abscess, surgical intervention, microbiologic results, antibiotic type and duration, and CRP values and time frame of CRP evaluation. We followed patients up for 6 months after treatment completion.

The inclusion criteria were treatment with either oral or parental antibiotic therapy for ≥6 weeks, a CRP value within 7 days of diagnosis, and ≥1 CRP value obtained after ≥1 week of treatment. All patients received parental antibiotic therapy during part of their treatment. Antibiotic treatment could extend beyond 6 weeks at the discretion of the treating infectious diseases physician. Patients could have other sites of infection, including epidural, psoas, or paraspinal abscess, endocarditis, skin and soft-tissue infection, and diabetic foot wounds. We included patients who underwent epidural abscess surgery or other surgical intervention as deemed adequate by surgical consultation to achieve source control. All surgery was performed during the index admission. The exclusion criteria were infected spinal hardware at any time during the treatment course, isolated facet joint septic arthritis, loss to follow-up, medication nonadherence, death within 6 weeks after treatment initiation, or NVO with *Brucella, Coxiella*, mycobacteria, or fungi.

### Study Outcomes

The primary outcome was treatment failure of antibiotic therapy assessed by an infectious diseases physician after completion of ≥6 weeks of therapy and within 6 months of the initial diagnosis. Treatment failure consisted of microbiologic or clinical failure. Microbiologic failure was defined as a positive blood or spinal tissue culture of the same or different organism after the end of treatment. Clinical failure was defined as significantly worsened spinal imaging as determined by the treating infectious diseases physician, new spinal abscess (epidural, psoas, or paraspinal muscle), resumption of antibiotics due to concern for an ongoing NVO, or death from a direct complication of NVO.

### Statistical Analysis Design

We used standard descriptive statistics to compare demographic and clinical data. Based on prior literature, and our univariate analyses, we designated *Staphylococcus aureus* infection and presence of an epidural abscess as risk factors for treatment failure [2, 3, 11, 12]. We deemed patients high risk if they exhibited one or both of these risk factors. All others were considered low risk.

We used Mann-Whitney rank sum univariate analyses to compare the first CRP value at diagnosis, the last CRP value while on treatment, and the percent difference between the first and last CRP values in patients who experienced treatment success versus those with treatment failure. We stratified these analyses by risk category. We also defined CRP cutoffs after graphic and quintile analyses to inform which CRP variables to enable χ^2^ comparisons between patients with treatment success versus treatment failure.

We conducted multivariate logistic analyses of the association between CRP variables and treatment failure while adjusting for *S aureus* infection and the presence of an epidural abscess in all patients as well as among those at high risk. We performed likelihood ratio testing for these multivariate models compared with the same models with interaction terms. All statistical analyses were conducted using Stata software, version 18.5 (StataCorp).

## RESULTS

**Figure 1.**
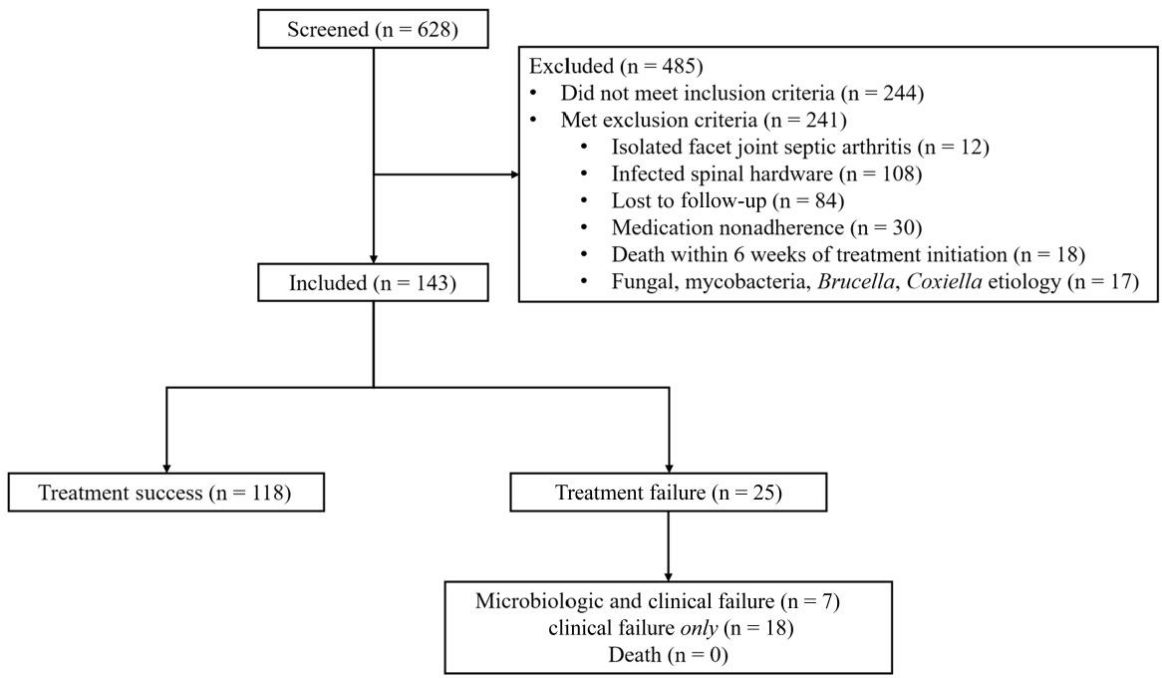
Flowchart of included patients. Some patients met >1 exclusion criterion.

Of the 628 patients with NVO identified by our initial screen, 143 met study inclusion criteria and were included in our final analyses (Figure 1). Demographic and clinical data are shown in Table 1. The mean patient age was similar in both groups, and a majority of patients were male. Sixty-seven patients were categorized as high risk. Of the 143 patients, 25 (17.5%) experienced treatment failure, 18 of whom experienced clinical failure alone in 18 and both microbiologic and clinical failure in 7. Treatment failure occurred in 18 high-risk patients (26.9%) and 7 low-risk patients (9.2%). Three patients with culture-negative NVO experienced treatment failure. One of them was subsequently found to have methicillin-resistant *S aureus* in a bone biopsy specimen. The other 2 cases remained culture negative at the time of treatment failure. There were no deaths related to NVO in the treatment failure group.

**Table 1.** Demographic and Clinical Descriptive Characteristics by Treatment Outcome

Characteristics	Patients, No. (%)^a^	
	Treatment Success	Treatment Failure
Total	118 (82.5)	25 (17.5)
Sex		
Male	80 (67.8)	15 (60.0)
Female	38 (32.2)	10 (40.0)
Race		
White	107 (90.7)	23 (92)
Nonwhite	11 (9.3)	2 (8.0)
Age, mean, y	60.5	59.7
Medical comorbid conditions		
Diabetes mellitus	27 (22.9)	7 (28.0)
Obesity	28 (23.7)	8 (32.0)
Rheumatoid arthritis	3 (2.5)	0 (0.0)
Chronic kidney disease	10 (8.5)	1 (4.0)
End-stage renal disease	0 (0.0)	1 (4.0)
Intravenous drug use	26 (22.0)	5 (20.0)
Cirrhosis	10 (8.5)	2 (8.0)
Malnutrition	2 (1.7)	0 (0.0)
Human immunodeficiency virus	0 (0.0)	0 (0.0)
Active cancer	2 (1.7)	2 (8.0)
Other immunocompromised state	5 (4.2)	0 (0.0)
Other rheumatologic disease	3 (2.5)	1 (4.0)
Spinal segment involved		
Cervical	15 (12.7)	2 (8.0)
Thoracic	26 (22.0)	7 (28.0)
Lumbar	88 (74.6)	19 (76.0)
Concurrent infectious syndrome		
Infective endocarditis	12 (10.2)	3 (12.0)
Epidural abscess	23 (19.5)	11 (44.0)
Psoas abscess	18 (15.3)	5 (20.0)
Paraspinal muscle abscess	6 (5.1)	3 (12.0)
Skin and soft-tissue infection	6 (5.1)	2 (8.0)
Diabetes mellitus foot ulcer	3 (2.5)	2 (8.0)
Blood culture obtained	114 (96.6)	25 (100.0)
Bacteria grown in blood culture(s)	64 (54.2)	16 (64.0)
MRSA	2 (1.7)	1 (4.0)
MSSA	26 (22.0)	11 (44.0)
Coagulase-negative staphylococci	7 (5.9)	0 (0.0)
Enterococci	2 (1.7)	0 (0.0)
β-Hemolytic streptococci	6 (5.1)	1 (4.0)
Viridans streptococci	6 (5.1)	2 (8.0)
Enterobacteraciae	6 (5.1)	1 (4.0)
Other	9 (7.6)	0 (0.0)
Spine culture obtained	72 (61.0)	10 (40.0)
Bacteria grown in spine culture(s)	45 (38.1)	8 (32.0)
MRSA	0 (0.0)	1 (4.0)
MSSA	16 (13.6)	3 (12.0)
Coagulase-negative staphylococci	6 (5.1)	2 (8.0)
Enterococci	4 (3.4)	0 (0.0)
β-Hemolytic streptococci	2 (1.7)	0 (0.0)
Viridans streptococci	6 (5.1)	0 (0.0)
*Cutibacterium*	1 (0.8)	0 (0.0)
Enterobacteraciae	8 (6.8)	2 (8.0)
*Pseudomonas aeruginosa*	1 (0.8)	1 (4.0)
Other	6 (5.1)	1 (4.0)

Abbreviations: MRSA, methicillin-resistant *Staphylocccus aureus*; MSSA, methicillin-sensitive *S aureus.*

^a^Data represent no. (%) patients unless otherwise specified.

The median time from diagnosis to treatment failure was 55 days. Patients who experienced treatment failure had more epidural abscesses and longer courses of antibiotics. The median duration of antibiotic therapy was 42 days for the treatment success and 46 days for the treatment failure group. The median interval between the first and last CRP values was 43 days (interquartile range, 39–49 days). Of note, surgical intervention without hardware placement for an epidural abscess or other related complication occurred in 14.4% of patients in the treatment success group and 20.0% in the treatment failure group. The rate of treatment failure in patient undergoing any surgery was 23.8%, compared with 18.9% for those not undergoing surgery.

**Figure 2.**
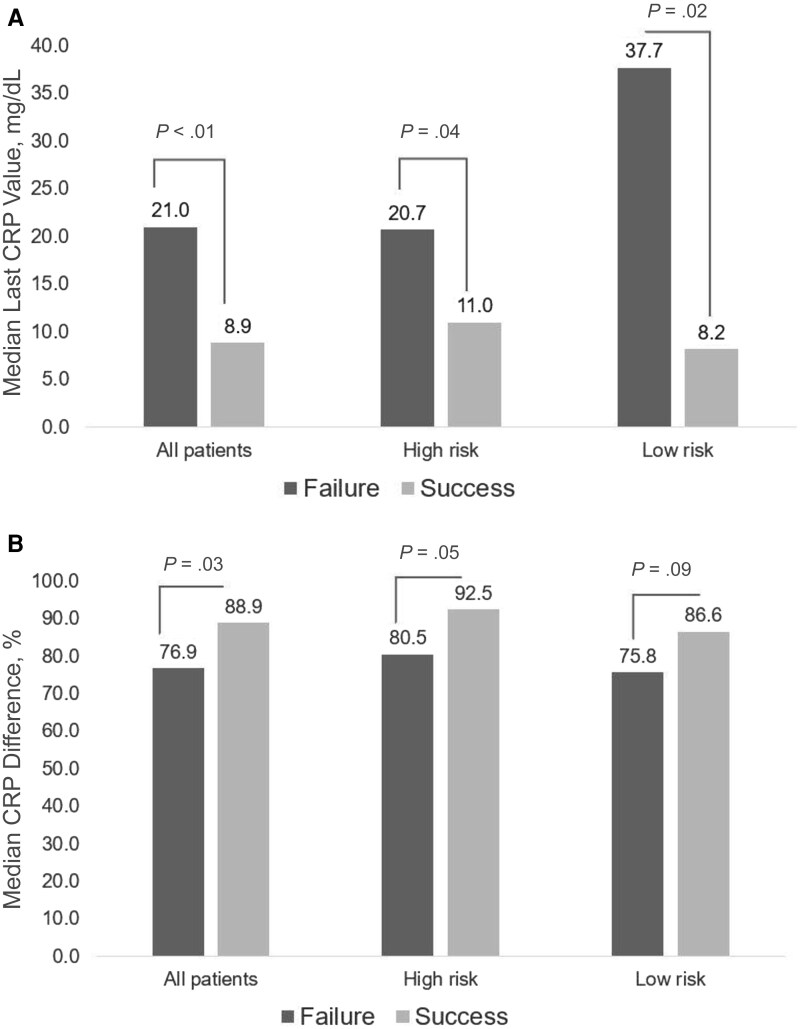
C-reactive protein (CRP) values per risk category by treatment outcome. *A*, Median last CRP value. *B*, Median CRP percent difference from first to last CRP value. Statistical analysis with Mann-Whitney rank sum testing was used to define *P* values.

Table 2 and Figure 2 depict univariate associations of CRP values with treatment failure. In univariate analyses, almost all CRP values were significantly higher among patients with treatment failure, including within the high-risk patient subgroup. The median percent CRP change from baseline to treatment exhibited only a borderline univariate association with treatment failure in the low-risk group.

**Table 2.** Univariate Analyses of the Association Between C-Reactive Protein Values or Risk Factors and Treatment Failure in Native Vertebral Osteomyelitis

CRP Value or Risk Factor	Treatment Success	Treatment Failure	*z* or χ^2^ Value	*P* Value
Median values				
First CRP value, mg/dL	122.4	161.1	*z* = −1.31	.19
Days from antibiotic start of last CRP	42.0	46.0	*z* = −1.77	.08
Last CRP value, mg/dL				
Overall	8.9	21.0	*z* = −3.27	<.01
High-risk group^a^	11.0	20.7	*z* = −2.08	.04
Low-risk group, mg/dL	8.2	37.7	*z* = −2.32	.02
CRP difference, %				
Overall	88.9	76.9	*z* = 2.13	.03
High-risk group^a^	92.5	80.5	*z* = 1.99	.05
Low-risk group	86.6	75.8	*z* = 1.7	.09
Risk factors, no. (%)				
Last CRP value >10 mg/dL	54 (75.0)	18 (25.0)	χ^2^ = 5.68	.02
Last CRP value >20 mg/dL	29 (67.4)	14 (32.6)	χ^2^ = 9.69	<.01
CRP % difference <80%	38 (71.7)	15 (28.3)	χ^2^ = 6.83	.01
Age ≥60 y	75 (84.3)	14 (15.7)	χ^2^ = 0.5	.48
*Staphylococcus aureus* infection	37 (71.2)	15 (28.8)	χ^2^ = 7.31	.01
Epidural abscess	23 (67.6)	11 (32.4)	χ^2^ = 6.84	.01
High risk^a^	49 (73.1)	18 (26.9)	χ^2^ = 7.69	.01

Abbreviation: CRP, C-reactive protein.

^a^High risk was defined as the presence of *S aureus* infection, epidural abscess, or both.

Unadjusted and adjusted multivariate logistic regression models are detailed in Table 3. While >1 unadjusted model suggested a borderline association between various CRP variables and treatment failure, most of these associations were attenuated markedly in adjusted models. The odds ratios (ORs) for all associations of CRP values with treatment failure closely approximated 1. The last CRP value and the CRP percent difference retained a borderline association with treatment failure in the high-risk cohort.

**Table 3.** Logistic Regression Analyses of the Association Between C-Reactive Protein Measurements and Treatment Failure in Native Vertebral Osteomyelitis

CRP Measurement	Association with Treatment Failure			
	Unadjusted Analysis		Adjusted Analysis	
	OR (95% CI)	*P* Value	OR (95% CI)	*P* Value
Last value	1.02 (1.00–1.04)	.02	1.02 (1.00–1.03)	.06
% Difference	0.99 (.99–1.00)	.19	0.99 (.98–1.00)	.11
Last value >10 mg/dL	1.01 (1.00–1.03)	.09	1.01 (.99–1.02)	.20
Last value >20 mg/dL	1.01 (1.00–1.02)	.19	1.01 (.99–1.02)	.36
% Difference <80%	1.00 (.99–1.02)	.66	1.01 (.99–1.03)	.37
Last value for high-risk group^a^	…	…	1.01 (1.00–1.03)	.10
% Difference for high-risk group^a^	…	…	0.98 (0.97–1.00)	.06

Abbreviations: CI, confidence interval; CRP, C-reactive protein; OR, odds ratio.

^a^High risk was defined by the presence of *Staphylococcus aureus* infection, epidural abscess, or both.

## DISCUSSION

NVO is associated with substantial morbidity and mortality rates and high rates of treatment failure. Traditionally, despite uncertain evidence, inflammatory markers like CRP have been used to predict which patients are likely to experience treatment failure and thus who might benefit from longer courses of antibiotic therapy [4–6]. Our study questions the value of this common practice by showing that there were only borderline statistical associations between any CRP value and treatment failure. Notably, while our univariate models suggested some association between CRP values and treatment failure, the strength of association weakened considerably with adjustment for well-established treatment failure risks of *S aureus* infection and/or epidural abscess. All ORs for such associations closely approximated 1, raising questions about whether this common practice truly adds clinical value, especially among low-risk patients.

The practice of evaluating CRP in the treatment of NVO is based largely on clinical experience and small retrospective studies. One of the earliest studies (from 1997) to assess inflammatory marker trends in NVO treatment response evaluated erythrocyte sedimentation rate (ESR) in 44 patients and showed that this marker was slow to change. In addition, most patients had a <50% decrease in ESR within the first month of treatment. The authors stated that the ESR response at 4 weeks of treatment was not a clear predictor of success, yet their study is commonly cited for the use of inflammatory markers in NVO [13]. Its findings have also been extrapolated to the use of CRP in evaluating treatment response for NVO. Further literature looked at a specific subset of patients with postoperative spinal wound infections and showed that patients with a decrease in CRP after 4 weeks of treatment were more likely to have clinical improvement [7]. The study was small, consisting of only 21 patients with postoperative wounds, of which 15 included hardware placement, and only 3 were felt to have a deep infection. In short, that study evaluated a much different patient population with postsurgical wounds that largely did not include osteomyelitis; however, it is cited in the Infectious Diseases Society of America (IDSA) guidelines as a reason to monitor CRP values for NVO [4, 7].

Another study showed that 62.5% patients with paravertebral or epidural abscess had improved magnetic resonance imaging findings 1 month into treatment despite ongoing elevations in CRP, underscoring the nonspecific nature of CRP values [14]. Yet another study found higher rates of treatment failure among patients with vertebral osteomyelitis who had a specific CRP value >2.75 mg/dL at the fourth week of antibiotic administration [9]; this was a small study with only 6 failures in a total cohort of 45 patients, of whom 36 underwent surgery. In addition, the analyses of CRP values and treatment failure were not controlled for *S aureus* or epidural abscess, as was done in our study. In contrast, in our larger study using multivariate analysis, we were unable to define any significant CRP threshold values that predicted treatment failure. Overall, this highlights the limited data available to guide the use of CRP in NVO management.

NVO treatment guidelines from the IDSA point out that it has not yet been possible to determine the appropriate frequency or utility, if any, of monitoring inflammatory markers for treatment of NVO [4]. Our data should deepen that ambivalence and raise the question of whether the costs of the practice are justified by added clinical value. Importantly, the French Society of Infectious Diseases recommends CRP monitoring for treatment of NVO, citing a retrospective review of 296 patients showing that CRP values decreased over the course of treatment in a fashion related to clinical resolution of infection. CRP was used due to a greater decrease in comparison to the trend of values for a white blood cell count [10, 15]. Our study also shows decreasing CRP values during treatment and only marginally greater CRP values among patients with treatment failure. These findings along with the close-to-1 ORs and borderline statistical significance of the association between these CRP metrics and treatment failure question the added clinical value of such measurements.

Results of the multivariate model associating last CRP with treatment failure approached but did not reach statistical significance (*P* = .06) when the high-risk factors of *S aureus* infection and epidural abscess were controlled for. There may be value in serial CRP monitoring specifically in this population, though our study did not prove it. This is an opportunity for further research. Evaluation with further multivariate models in the high-risk patient groups showed no or borderline statistical significance for the last CRP value, the CRP percent difference, or different threshold values of CRP. Notably, *S aureus* infection and epidural abscess remain clinically significant and continue to have a higher OR for treatment failure.

Our study's strengths includes its being one of the largest studies of whether CRP predicts treatment failure in NVO, its multisite origin, and adjustment for potential confounders of the association between CRP and treatment failure. Its major limitation is its retrospective design, which could introduce selection bias or other forms of bias. There was also no standardized follow-up protocol because treatment duration was chosen at the discretion of the treating clinician, potentially decreasing the study’s power to detect associations between CRP and treatment failure. That said, the clinical value of CRP assessment cannot be strong if only detectable outside of real-life clinical practice. In addition, our study's generalizability may have been affected by the relative sex and racial homogeneity, as well as the large dropout rate among screened patients. This large exclusion population also reflects the challenge in retrospectively studying a heterogenous disease process. Treatment duration was longer among patients who experienced treatment failure, perhaps suggesting clinician recognition that those patients were at risk for treatment failure. Our ascertainment of treatment success versus failure until 6 months from treatment initiation has a low but nonzero chance of missing very late treatment failure, but we believe the low likelihood of relapse several months after treatment discontinuation is unlikely to alter the low clinical utility of checking CRP at about 6 weeks of treatment.

The association of CRP metrics with treatment failure could be confounded by concomitant inflammatory states, such as *Clostridioides difficile* colitis, central vascular catheter infection, or coincidental rheumatologic conditions. We chose not to include these coincidental clinical conditions in our multivariate model since our goal was not to provide a perfect estimate of the association between CRP measurements and NVO treatment failure but rather to determine whether measuring CRP helps clinicians predict treatment failure. We suspect that conducting a multivariate logistic regression model at the bedside may be outside the scope of most clinicians’ practice.

Despite its being among the largest studies of the relation between CRP and NVO treatment failure, this study's small sample size raises the possibility that it was underpowered to detect subtle associations between CRP and treatment outcome. The very subtlety of any possibly missed associations again argues against the great clinical utility of measuring CRP in patients with NVO.

In conclusion, CRP values are weakly associated with treatment failure in NVO in multivariate analyses adjusted for infection with *S aureus* or the presence of an epidural collection. This finding questions whether the common clinical practice of measuring CRP in patients with NVO truly adds clinical value.
