# Correction to: Using C-Reactive Protein Measurements to Predict Treatment Failure in Native Vertebral Osteomyelitis: A Multisite Retrospective Cohort Study

**DOI:** 10.1093/ofid/ofag355

**Published:** 2026-06-09

This is a correction to: Brendan Campbell, Tim Lahey, Andrew J Hale, Using C-Reactive Protein Measurements to Predict Treatment Failure in Native Vertebral Osteomyelitis: A Multisite Retrospective Cohort Study, *Open Forum Infectious Diseases*, Volume 13, Issue 5, May 2026, ofag239, https://doi.org/10.1093/ofid/ofag239

In the originally published version of the paper there was a typographical duplication of the last two words of the title. This has now been corrected.

